# Supplementary material for: A Genome-Wide Alternative Splicing Analysis of Gossypium arboreum and Gossypium raimondii During Fiber Development
Source: Plants (Basel). 2024 Oct 8;13(19):2816. doi: 10.3390/plants13192816 (PMC11479146; doi:10.3390/plants13192816)
Supplement: Supplementary file 1 [file plants-13-02816-s001.zip › Supplementary Figures.pdf]

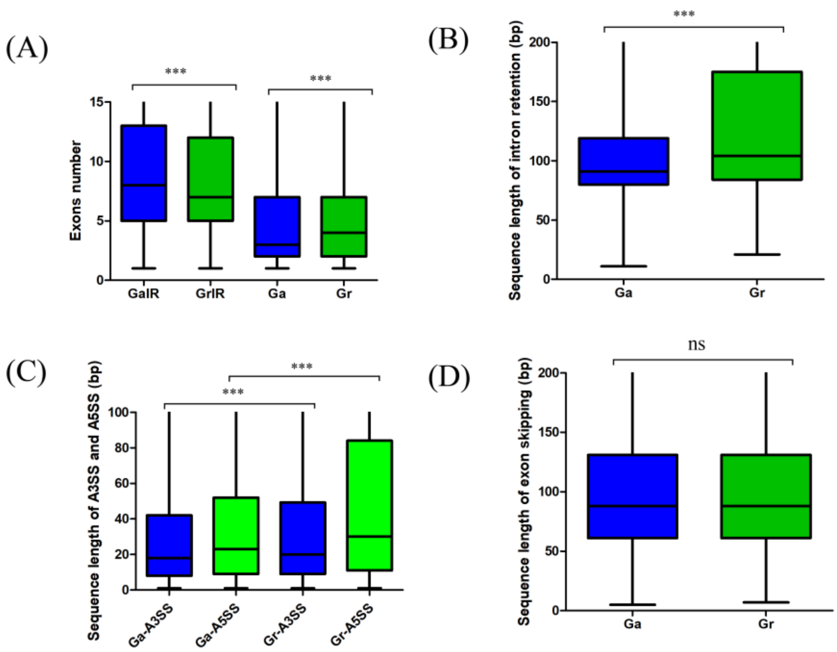

Figure S1. Exon number and length of four basic alternative splicing events. (A): The exon number of AS genes and expressed genes. The AS length of (B): IR; (C): A3SS and A5SS; (D): SE. (*** P < 0.001, by Student's t-test)

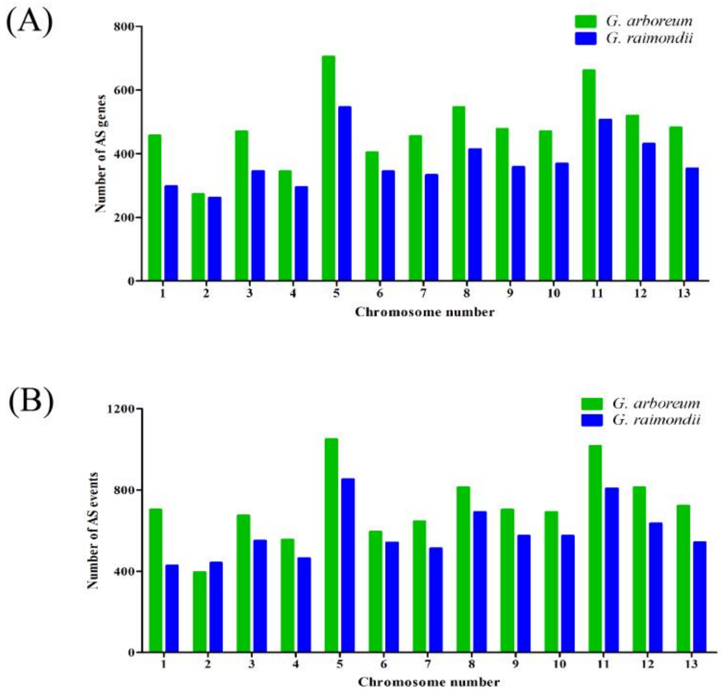

Figure S2. Statistics of AS genes and AS events in the chromosomes identified in *G.arboreum* and *G.raimondii*. (A): AS genes, (B): AS events.
